# A Global Initiative for Chronic Obstructive Lung Disease (GOLD) Science Committee perspective on exacerbations and cardiovascular risk in chronic obstructive pulmonary disease

**DOI:** 10.1093/ajrccm/aamag231

**Published:** 2026-05-08

**Authors:** Dave Singh, Alvar Agustí, Jean Bourbeau, Fernando J Martinez, David M G Halpin, Claus F Vogelmeier

**Affiliations:** Respiratory Research Group, University of Manchester, Manchester University NHS Foundation Trust, Manchester, United Kingdom; Medicines Evaluation Unit an IQVIA Business, Manchester, United Kingdom; Pulmonary Department, Catedra Salud Respiratoria, University of Barcelona, Respiratory Institute, Clinic Barcelona, FCRB-IDIBAPS, CIBER Enfermedades Respiratorias, Barcelona, Spain; McGill University Health Centre, Division of Clinical and Translational Research, McGill University, Montreal, QC, Canada; Department of Medicine, UMass Chan Medical School, Worchester, MA, United States; Faculty of Health and Life Sciences, University of Exeter Medical School, University of Exeter, Exeter, United Kingdom; Department of Medicine, Pulmonary and Critical Care Medicine, University Hospital Giessen and Marburg, German Center for Lung Research (DZL), University of Marburg, Marburg, Germany

## Introduction

It is well established that cardiovascular diseases (CVDs), including coronary artery disease, heart failure, and arrhythmias, among others, are highly prevalent in patients with chronic obstructive pulmonary disease (COPD).^1^^,^^2^ This not surprising as COPD and CVD share common risk factors such as cigarette smoking and increasing age.^3^ However, there are mechanistic interactions between these 2 conditions that make COPD an independent risk factor for the development of CVD after accounting for confounding variables.^1^

Patients with COPD often suffer episodes of exacerbation of their symptoms, which impact health status and worsen their prognosis.^4^^,^^5^ Several studies have now clearly shown that the risk of suffering a cardiovascular (CV) event and/or death is increased following an exacerbation and remains elevated for several months after the exacerbation.^6-10^ These observations reinforce the importance of preventing the occurrence of exacerbations and raise relevant questions about the optimal management during and after them to prevent CV events.^11^ The Global Initiative on Obstructive Lung Disease (GOLD) report 2025 highlighted the interactions between exacerbations and CV risk, with further changes in the GOLD 2026 report to update the evidence and discuss the clinical implications.^12^ This perspective from the GOLD Science Committee focuses on the increased CV risk during and after an exacerbation, reviewing the evidence, discussing how to better address this in clinical practice, and identifying evidence gaps that could be addressed by future research.

## A syndemic perspective

The term *syndemic* has been used to describe the situation where different diseases share risk factors and mechanisms and is now being increasingly used to describe the relationship between COPD and CVD (Figure 1).^3^^,^^11^ As the lungs and heart have close anatomic and physiologic interactions, dysfunction of 1 of these 2 organs often causes a problem for the other; for example, left ­ventricular failure causes pulmonary edema, while pulmonary hypertension causes right ventricular hypertrophy. Furthermore, systemic inflammation is a feature of COPD and CVD, with high levels of biomarkers such as C-reactive protein (CRP) and fibrinogen associated with increased risk of both exacerbations and CV events.^13^^,^^14^ The syndemic occurrence of COPD and CVD, coupled with the close anatomical and functional relationship between these organ systems, causes a high prevalence of CVD in COPD patients. Consequently, CVDs are a common cause of death in COPD patients, particularly in those with mild-moderate disease where respiratory failure is less common.^15^ Likewise, COPD (often underdiagnosed, hence undertreated) is prevalent in patients with CVD but may not be diagnosed or treated optimally,^16^

Chronic obstructive pulmonary disease is an independent predictor for CVD. A retrospective cohort analysis from Canada reported a 25% increase in the rate of major adverse CV events (MACE) in COPD patients without a history of CV vs matched individuals without COPD after adjusting for CV risk factors.^17^ Data from the United Kingdom showed that the CV risk score in COPD patients calculated using QRISK3 underestimated the risk of developing CVD, implicating COPD itself as a risk factor.^18^ Using UK Biobank data (*n* = 308 415), abnormal spirometry including airflow obstruction predicted the incidence of severe CV events such as myocardial infarction and stroke.^19^ Although COPD was not specifically identified in this latter analysis, it highlights that poor lung function is associated with increased CV risk. Supporting this observation, a longitudinal study in the Canadian Cohort of Obstructive Lung Disease found that individuals with COPD or preserved ratio with impaired spirometry were at higher risk to develop CVD compared with individuals with normal spirometry.^20^ Furthermore, the Multi-Ethnic Study of Atherosclerosis (MESA) study conducted in individuals aged 45-84 years and without a history of CVD (*n* = 6814) reported that lower FEV_1_ was associated with an increased risk of developing atrial fibrillation.^18^

## Epidemiological evidence: exacerbations and CV events

Multiple studies have documented an increased risk of acute CV events after the onset of an exacerbation. An analysis of 213 466 COPD patients in the United Kingdom reported an increase in CV events, encompassing acute coronary syndrome, arrhythmia, heart failure, ischemic stroke, and pulmonary hypertension after an exacerbation.^7^ For severe exacerbations (requiring hospitalization), the risk was highest in the 2 weeks immediately following the onset of the exacerbation (adjusted hazard ratio [HR] = 14.5), while the increased risk following a moderate exacerbation was of a lower magnitude and showed a later peak at 14-30 days (adjusted HR = 1.93). The risk of CV events subsequently declined over time but was still raised at 1 year and beyond. Analysis of COPD cohort data in Canada (*n *= 142 787) reported a similar pattern of results using a slightly different composite endpoint of all-cause death or a first hospitalization for acute coronary ­syndrome, arrhythmia, heart failure, or cerebral ischemia; the incidence of a composite event was increased from days 1 to 7 following exacerbation onset (adjusted HR = 15.86) with a decline thereafter but again remaining elevated at 1 year, while the risk was greater for severe vs moderate exacerbations.^6^ Increased risk was observed for all components of the composite, with decompensation of heart failure showing the greatest increased risk as the hazard ratio reached 72.4 between days 1 and 7. Heart failure was also an important component outcome with greater risk in the UK study, with the hazard ratio being 2.87 from days 1 to 14. These cohort studies highlight the influence of exacerbations on different CV outcomes, with increased risk observable at exacerbation onset and remaining elevated over time. Some older ­studies of CV outcomes after exacerbation did not report heart failure or arrhythmias.^21^^,^^22^ Nevertheless, a meta-analysis including 533 672 COPD patients from such studies reported an increased risk of myocardial infarction (risk ratio = 2.43) and stroke (risk ratio = 1.68) within the time period of 1-3 months after an exacerbation.^9^

An analysis of a COPD cohort in Germany (*n* = 126 795) used the same composite definition as in the Canadian cohort already described,^8^ and in line with their results, it also found that the highest increased risk observed was between days 1 and 7 (HR = 8.6) and declining thereafter but still elevated at 1 year, while greater risk was observed with severe vs moderate exacerbations. Among individuals who were identified with a new diagnosis of COPD (36.6% of the cohort), there was an increase in the rate of a composite event after the first exacerbation, which was further increased after a second exacerbation, while the results for a second or third exacerbation were similar. These observations suggest an accumulation of CV risk over time in newly diagnosed COPD patients who suffer from repeated exacerbations.

A pooled analysis included the studies from Canada^6^ and Germany^8^ already described while adding cohorts from other countries that used a common protocol and composite definition (as was used in the Canadian study).^23^ This provided a large sample size (*n* = 1 030 875) to understand the magnitude and duration of increased risk. Within the 7 days after exacerbation onset, the risk of a composite event was increased by approximately 10-fold and remained increased for up to 1 year. While the risk was higher for a severe exacerbation, the risk after a moderate exacerbation was increased approximately 2-fold up to day 14 and remained elevated for up to 6 months.

## Mechanisms of increased CV risk during and after an exacerbation

There are several, nonmutually exclusive mechanisms that can underlie the increase in CV risk during and after an exacerbation (Figure 1).^24^^,^^25^ First, systemic inflammation is common in COPD and CVD^25-27^ and increases further during exacerbation with increased levels of IL-6, which acts in the liver to upregulate the production of acute phase proteins including CRP and fibrinogen.^28^^,^^29^ There may also be increased levels of systemic oxidative stress and an increase in circulating leukocytes, endothelial dysfunction, platelet activation, and coagulation disorders; these factors combine to cause plaque instability with rupture or erosion of the fibrous cap thereby increasing the risk of thrombus formation.^25^^,^^30^ Second, narrowing of the airways during an exacerbation, caused by mechanisms such as airway inflammation, mucus obstruction, or smooth muscle constriction, can cause increased gas trapping and lung hyperinflation.^24^^,^^31^ This increases intrathoracic pressure, resulting in compression of blood vessels and the heart. This can reduce venous return and cause atrial compression, thereby limiting the preload volume. Compression of the pulmonary vessels and ventricles can reduce cardiac output.^31^ Finally, impaired pulmonary gas exchange during exacerbations, with ventilation/perfusion mismatching contributing, can cause or worsen arterial hypoxemia.^31^ A combination of reduced cardiac output and arterial hypoxemia can jeopardize myocardial oxygenation. These multiple mechanisms may be present to varying degrees in different COPD patients during the stable state and then worsen further during exacerbation.

Studies involving computed tomography (CT) scanning or lung volume assessment have shown that increased emphysema or hyperinflation is associated with reduced left ventricular filling volumes and lower cardiac output,^32^^,^^33^ with lower left ventricular filling volumes associated with increased mortality.^34^ These ­findings demonstrate the impact that a specific pulmonary ­characteristic (emphysema) has on cardiac function. The MESA study measured segmental airway wall area (WA) and percent ­emphysema by CT and cardiac ventricle parameters by magnetic resonance imaging in COPD patients without CVD.^35^ During 6 years longitudinal follow-up, it was observed that greater WA at baseline was associated with greater increases in right ventricular mass. While greater emphysema at baseline was associated with lower left ventricular filling and cardiac output, this did not worsen over time. These findings highlight that different pulmonary manifestations of COPD may alter cardiac function in different ways.

## Clinical implications

The presence of underlying CVD should be investigated in all clinically stable COPD patients, given the syndemic occurrence and the ability of each disease to contribute to the other (Figure 1). This assessment should be done as early as possible after diagnosis. Importantly, established CVD risk scores in the general population, including Framingham or Qrisk3, underestimate the risk in COPD patients.^18^ To overcome this limitation, the recently developed Qrisk4 score includes COPD as a risk factor for developing CVD.^36^ A recent consensus statement on this topic highlighted that the identification of CVD (in COPD patients) needs to be cost-effective and accurate.^2^ The measurement of natriuretic peptides is a well-accepted method for evaluating heart failure. Computed tomography scans performed for the clinical assessment of COPD or lung cancer screening offer an opportunity for CVD screening, for example by the identification of coronary artery calcification.

The presence of CVD in COPD patients should be treated according to current guidelines. There has been interest in the use of β-blockers and statins to improve COPD outcomes, but clinical trials in COPD patients who did not have the appropriate CV indication for these treatments have been negative.^37-39^ These treatments should be prescribed according to the presence of the appropriate CV indication in COPD patients.

Clinical trials in COPD patients with increased exacerbation risk have shown that single inhaler triple therapy containing an inhaled corticosteroid (ICS) plus long-acting β_2_ agonist (LABA) plus long-acting muscarinic antagonist (LAMAs) reduces exa­cerbations compared to LABA/LAMA treatment; this benefit of the ICS component is an approximately 25% exacerbation rate ­reduction.^40-42^ Two of the largest single inhaler triple therapy studies were conducted in COPD patients with high exacerbation risk (≥2 moderate exacerbations or 1 severe exacerbation in the previous year despite maintenance inhaled treatment); the exacerbation reduction with ICS was accompanied by a relative reduction in mortality up to 50%.^43^^,^^44^ These mortality analyses were predefined endpoints and reached statistical significance, although the significance was either nominal due to hierarchical testing or not adjusted for multiplicity. As expected, deaths due to CV causes were common in these studies; interestingly, ICS treatment numerically reduced these events. A potential mechanism is that the ICS-related prevention of exacerbations contributes to a decrease in CV events and mortality.

The benefit of ICS on exacerbations in these triple therapy studies was greater in patients with higher blood eosinophil counts (BECs),^45^ while the effect of ICS on mortality was also greater at higher BECs in the The Efficacy and Safety of Triple Therapy in Obstructive Lung Disease (ETHOS) study.^44^ These observations are explained by the fact that higher BECs are associated with increased levels of pulmonary type 2 inflammation^46^ and ICSs target pulmonary type 2 inflammation;^47^ therefore, ICSs have greater anti-inflammatory effects at higher BECs, which translates into greater exacerbation prevention.^45^ It has also been proposed that eosinophils themselves can act as drivers of atherosclerosis and thrombosis,^48^ providing an alternative mechanism by which ICSs provide clinical benefit (ie, by targeting type 2 inflammation and thereby lowering eosinophil activity related to CV risk).

A post hoc analysis of the ETHOS study investigated time to first MACE and time to first cardiac adverse event, demonstrating ICS treatment benefits for both outcomes.^49^ A post hoc analysis of the InforMing the PAthway of COPD Treatment (IMPACT) study focused on the association between ECG abnormalities, including the Cardiac Infarction Injury Score (CIIS), and clinical outcomes.^50^ The CIIS assesses myocardial injury, with higher scores being prognostic for increased mortality in COPD patients. Higher CIIS scores in the IMPACT study were associated with increased rates of CV events, exacerbations, and mortality. Furthermore, a positive benefit of ICS on CV events and mortality was only observed in the subgroup with higher CIIS scores. Taken together, these analysis from ETHOS and IMPACT suggest a benefit of ICS treatment on CV outcomes in COPD patients with high exacerbation risk, with the benefit being increased in patients with greater myocardial dysfunction. In contrast, other populations with lower exacerbation risk have not reported positive results. For example, the Study to Understand Mortality and Morbidity in COPD included COPD patients with increased risk of CV events but low exacerbation risk and reported no protective effect of ICS/LABA on all-cause mortality.^51^ Additionally, a retrospective analysis of UK primary care data (*n *= 113 353) showed no reduction in MACE events with ICS treatment in an unselected COPD population.^52^

The possibility that optimization of inhaled pharmacological treatment to prevent exacerbations can reduce CV risk opens a new window into the prevention of CV events in COPD patients. Further research is needed to understand the benefits of other COPD pharmacological interventions that prevent exacerbations on CV outcomes, for example biological treatments administered systemically (Table 1).

Finally, for COPD patients with severe exacerbations being assessed in a hospital setting, it would seem appropriate to measure CV biomarkers including troponin and brain-natriuretic peptides given the high CV risk documented in this situation. For moderate exacerbations, there is increased CV risk albeit lower in magnitude,^6-8^ so measuring CV biomarkers may be more relevant to individuals with a prior history of CVD. Evidence is needed to develop optimal strategies for detecting CV events during and after an exacerbation (Table 1). Additionally, research is needed to elucidate whether the routine use of preventive CV treatment (eg, aspirin, statins, blood pressure–lowering drugs) after an exacerbation is beneficial.

## Conclusion

Chronic obstructive pulmonary disease and CVD are intimately related with shared risk factors and mechanisms (ie, a syndemic origin).^1^^,^^2^ Cardiovascular events contribute significantly to the morbidity and mortality associated with COPD exacerbations, and the risk of these events remains elevated for many weeks after an exacerbation.^6-9^^,^^21^^,^^53^ Cardiovascular events occur in patients with COPD who have not previously been diagnosed with CVD, and conventional CV risk tools have historically underestimated the risk of CV events in patients with COPD.^36^^,^^54^ All patients with COPD should be investigated for occult CVD,^2^ and markers of CV risk should be assessed at the time of COPD exacerbations. Improving survival in COPD depends on reducing the risk of exacerbations, particularly severe exacerbations, addressing identified CV risk factors such as hypertension and hyper­lipidemia, and managing CVD when identified according to guidelines.^2^ Adopting a holistic approach to these syndemic ­conditions is key to improving outcomes for patients with COPD.^3^

## Supplementary Material

aamag231_supplementary_data

## Figures and Tables

**Table 1:** Research priorities to reduce cardiovascular (CV) risk associated with exacerbations.

**Investigate the effects of chronic obstructive pulmonary disease treatments on the reduction of CV events during and after an exacerbations (eg, biologic treatments).**
**Investigate the potential benefits of interventions with preventative treatments that target the CV system during and after an exacerbation.**
**Develop optimal clinical strategies for monitoring CV system (eg, using biomarkers) to detect CV events after an exacerbation. Such strategies may differ for severe vs moderate events.**

**Figure 1:**
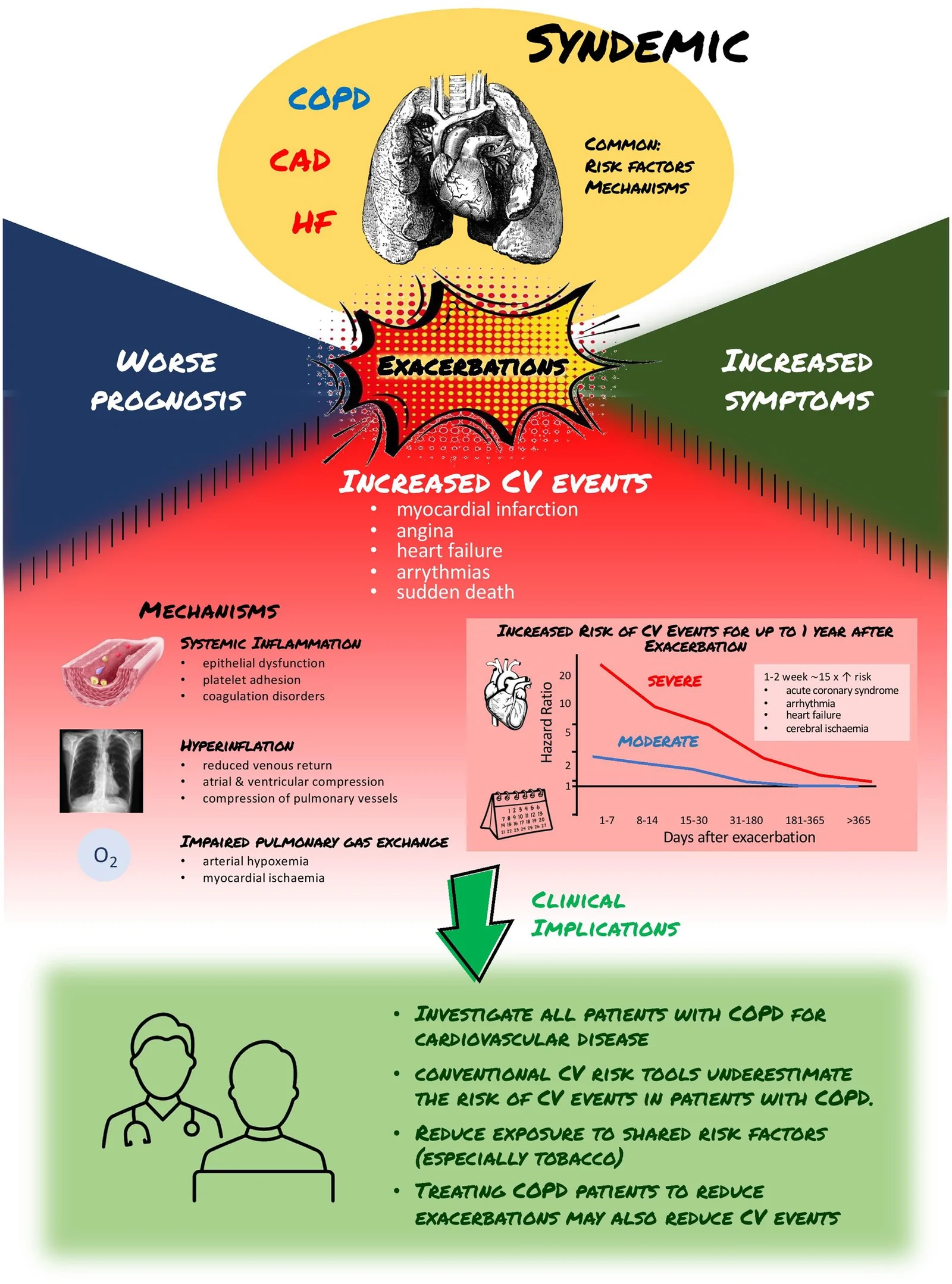
The role of exacerbations in provoking cardiovascular (CV) events in COPD patients. Graphic showing the relationship between CV risk and time after exacerbation onset uses information^6^^,^^7^^,^^8^^,^^9^^,^^10^^,^^23^ to visualize the approximate hazard ratio.
